# The influence of melatonin supplementation on *in vitro* culture of murine embryos from polycystic ovary syndrome experimental models

**DOI:** 10.5935/1518-0557.20250164

**Published:** 2026

**Authors:** Marcela de Oliveira Pinheiro, Fernanda Bertuccez Cordeiro, Giuliana Camila Ramirez Ramos, Dóris Ferreira Moriyama, Gabrielle Ferrante Alves de Moraes, Karla Pacheco de Melo, Maurício de Rosa Trotta, Fernando Prado Ferreira, Edson Guimarães Lo Turco

**Affiliations:** 1 Department of Surgery, Division of Urology, Human Reproduction Section, São Paulo Federal University - UNIFESP, São Paulo, 04039-060, Brazil; 2 Laboratory for Biomedical Research, Faculty of Life Sciences, Escuela Superior Politécnica del Litoral (ESPOL) Guayaquil, O90211, Ecuador; 3 CEDEME, Sao Paulo Federal University - UNIFESP, São Paulo, 04039-060, Brazil; 4 Department of Gynecology, Sao Paulo Federal University - UNIFESP, São Paulo, 04039-060, Brazil

**Keywords:** polycystic ovary syndrome, blastocyst, murine embryo development, melatonin supplementation, apoptosis, TUNEL assay

## Abstract

**Objective:**

Polycystic ovary syndrome (PCOS) negatively impacts oocyte and embryo quality. However, melatonin supplementation in assisted reproduction may enhance oocyte and embryo quality. This study aimed to analyze the effect of melatonin supplementation in embryo culture, by assessing embryos quality and development in murine models.

**Methods:**

C57BL/6J mice strain were divided into 6 groups: PCOS; PCOS with melatonin; placebo; placebo with melatonin; controls; and controls with melatonin. Embryo classification was performed during all developmental phases. TUNEL assay analysis was carried out in blastocysts.

**Results:**

The melatonin supplementation showed a positive influence during cleavage and morula development for all groups. The blastocyst rate was lower in the PCOS group when compared to placebo and controls. For the TUNEL assay, placebo and control groups supplemented with melatonin had a lower number of apoptotic cells compared to their respective non-supplemented groups.

**Conclusions:**

Melatonin supplementation exerts a beneficial impact on cleavage and morula development for all groups. For PCOS, the poor blastocyst quality and high apoptosis rate emphasizes the inherent challenges associated with this condition. Melatonin potentially mitigates apoptotic events during early embryo development, suggesting its relevance as a supplementary therapeutic approach.

## INTRODUCTION

Polycystic ovary syndrome (PCOS) is a complex endocrine disorder that commonly affects women of reproductive age (Ehrmann, 2005). diagnosis is established when at least two of the following criteria are met: oligo and/or anovulation; clinical and/or biochemical signs of hyperandrogenism; and polycystic morphology of ovaries at ultrasound (Fauser et al., 2012). PCOS is frequently accompanied by metabolic disturbances such as insulin resistance, glucose intolerance, and dyslipidaemia, besides an increased risk of hypertension, cardiovascular disease and psychological complications including depression and social stress (Dunaif, 2006; Xie et al., 2021).

A major concern in PCOS is the impairment of oocyte and embryo quality. Anovulatory infertility is a common clinical manifestation and a frequent indication for assisted reproductive techniques (ART). Although controlled ovarian stimulation in PCOS patients often results in a high number of follicles, the proportion of competent oocytes remains low (Lizneva et al., 2016; Da Broi et al., 2018). These typically show reduced capacity for meiotic maturation, fertilization, embryo development, and successful implantation (Da Broi et al., 2018).

Recent studies highlight the role of melatonin in reproductive function, largely due to its antioxidant and anti-apoptotic properties (Sun et al., 2020; Zhang et al., 2020). While primarily known as a regulator of circadian rhythms, melatonin is also produced in smaller quantities in various tissues, including the ovaries and placenta (Stefulj et al., 2001; Arendt & Skene, 2005; Sanchez-Hidalgo et al., 2009; Acuña-Castroviejo et al., 2014). In the ovaries, melatonin contributes to delaying ovarian aging through reduction of oxidative stress and modulation of autophagy (Tamura et al., 2017). In placentas, melatonin enhances nutrient transport and vascular dynamics on the uterine-placental interface (Chuffa et al., 2019).

At the cellular level, oocyte-granulosa cell communication is essential for oocyte maturation. Melatonin supplementation in granulosa cells increases levels of NADPH and glutathione, which may rescue oocytes from aging-related deficiencies and improve embryo quality (Zhang et al., 2022). Furthermore, the use of melatonin in ART has been associated with higher fertilization rates and blastocyst formation (Tamura et al., 2022). This treatment alters the transcriptome of granulosa cells, leading to an inhibition of cell death and promoting steroidogenesis and angiogenesis (Tamura et al., 2022).

Emerging evidence suggests melatonin may also have therapeutic benefits in PCOS, although underlying mechanisms remain unclear (Xie et al., 2021). In PCOS models, melatonin appears to regulate autophagy via the PI3k-kt pathway, reducing mitochondrial damage in oocytes (Zheng et al., 2021). In clinical settings, oral melatonin supplementation has been associated with improvement of oocyte and embryo quality in women undergoing IVF (Pacchiarotti et al., 2016).

Therefore, this study investigated the effects of melatonin supplementation in the embryo culture medium on the development and quality of embryos from PCOS murine models, providing new insights into melatonin’s influence on embryo morphology and its anti-apoptotic effects.

## MATERIAL AND METHODS

In this study, we used C57BL/6J mice of both female and male lineages. The cohort included 18 female mice aged between 6 and 10 weeks and 12 male mice aged between 12 and 18 weeks for the purpose of offspring generation. These animals were housed at the Center for the Development of Experimental Models for Medicine and Biology from the Federal University of São Paulo (UNIFESP). Following mating, female offspring were specifically chosen for the induction of polycystic ovary syndrome (PCOS) followed by the production of embryos. The study encompassed a total of 90 newborns, which were categorized into six groups (n=15 per group). All animals were maintained at a temperature of 21oC (± 2oC), subjected to a 12-hour light/dark cycle, and granted ad libitum access to food and water. Approval for this study was obtained from the Ethics Committee on the Use of Animals from UNIFESP under protocol number 9434210218.

**Figure 1:**
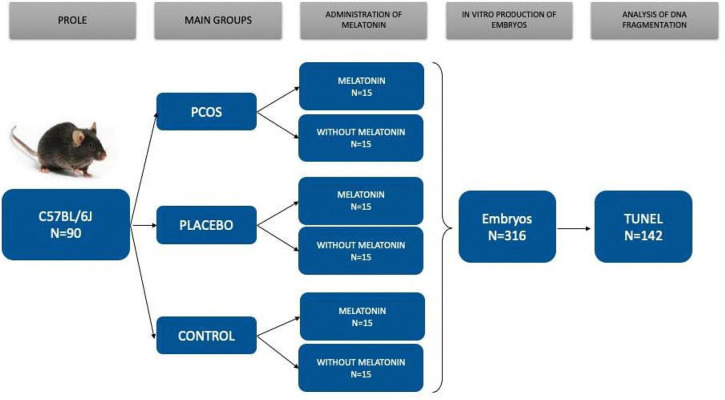
Experimental design workflow. From 316 Embryos produced in vitro, 142 embryos reached the blastocyst stage and were analyzed by TUNEL assay.

The groups were stratified based on the induction of PCOS and the administration of melatonin, as illustrated in Figure 1: (i) PCOS induction with melatonin administration group (PCOS-M); (ii) PCOS without melatonin group (PCOS); (iii) Placebo with melatonin administration group (PLA-M); (iv) Placebo without melatonin group (PLA); (v) Control group with melatonin administration (CONT-M); and (vi) Control group without melatonin administration (CONT). All female mice underwent ovarian stimulation and were paired with males for the production of embryos. A total of 316 embryos were generated, and their development was monitored until reaching the blastocyst stage. Additionally, the Terminal Deoxynucleotidyl Transferase dUTP nick end Labeling (TUNEL) assay was conducted to assess DNA fragmentation.

### PCOS induction

In the experimental induction of polycystic ovary syndrome (PCOS) in female mice, a meticulous procedure was followed. Specifically, 20µL of testosterone cypionate (EMS, Brazil) was subcutaneously injected into the dorsal region of offspring belonging to groups PCOS-M and PCOS on days 1 and 2 post-birth (Nohara et al., 2011). For the placebo groups, a comparable volume of 20µL of peanut oil was administered subcutaneously into the dorsal region of offspring from groups PLA-M and PLA during the same two-day period. The control groups did not receive any substance during this experimental process, ensuring a clear distinction between the treatment and control conditions.

### Ovarian stimulation and embryo production

Upon reaching reproductive age, specifically between 28 and 42 days, all female mice underwent a superovulation protocol to enhance the yield of oocytes. The protocol involved the intraperitoneal administration of 5IU of pregnant mare’s serum gonadotrophic (MSD, Brazil). Subsequently, 42 to 48 hours later, another 5IU of equine chorionic gonadotrophin (MSD, Brazil) was administered. Following this hormonal treatment, the females were paired with fertile adult males aged between 12 and 18 weeks.

After a period of 18 to 24 hours post-coitus, the females were sedated using a solution consisting of 20% isoflurane and 80% propylene glycol, followed by euthanasia through cervical dislocation. The uterine tubes were carefully collected and immediately immersed in HTF medium modified with HEPES (Irvine, USA). The uterine tubes were meticulously washed and cleaned, and any zygotes identified were denuded to remove cumulus cells. Subsequently, the zygotes were transferred to a plate containing CSCM-C medium, the surface of which was covered with mineral oil, to facilitate their development. This comprehensive process aimed to ensure optimal conditions for embryonic development and outcome evaluation.

### PCOS model proof

Following euthanasia, the ovaries’ histological specimens were immersed in 10% neutral buffered formalin for overnight fixation. A meticulous procedure ensued, involving dehydration in a graded series of ethanol, clearing with xylene, and embedding in paraffin. Each ovary underwent sectioning into a series of 4 µm slices, which were then affixed onto slides and subjected to staining with hematoxylin and eosin staining. The stained slides were examined under an optical microscope at 40x magnification, enabling the quantification of follicles per ovary. This histological analysis aimed to provide insights into ovarian morphology and support a detailed assessment of follicular development and distribution.

### Embryo culture and morphological analysis

The embryos were cultured in drops of 100 µL of CSCM-C continuous culture medium (Irvine, USA) covered with approximately 8mL of mineral oil (Irvine, USA). The supplementation of 10-6 mol/L of melatonin (Merck, USA) was performed according to group allocation, and the embryos were incubated at 37°C, 6% of CO2, and 90% of humidity for 96 hours until they reached the blastocyst stage.

The morphological analysis of the embryos was performed at 24h of culture (cleavage stage), followed by a second evaluation at 72h of culture (morula stage), and at 96h of culture (blastocyst stage) by using an inverted optical microscope (Olympus, Japan). Initial blastocyst analysis considered the degree of expansion of the blastocoel, the inner cell mass, and the trophectoderm (Gardner et al., 2000).

For statistical analysis, blastocysts were then classified using the criteria of the Society of Assisted Reproductive Technology (Heitmann et al., 2013), as good, fair, or poor. The categorical variables in this analysis were transformed into numerical variables to facilitate statistical analysis.

### TUNEL Assay

To assess DNA fragmentation in blastocysts, the TUNEL technique was employed, utilizing the In Situ Cell Death Detection Kit, Fluorescein (Roche, Switzerland). This assay detects fragmented DNA through labeling the terminal deoxynucleotidyl transferase-mediated dUTP nick-end labeling (TUNEL) method. Fluorescein-labeled nucleotides bind to the exposed DNA ends, enabling the visualization and quantification of DNA fragmentation. This crucial analysis offers insights into the integrity of genetic material, providing valuable information on the embryos’ developmental potential.

### Statistical Analysis

Statistical analysis was conducted using PASW 18.0 software (SPSS, Chicago, IL, USA). Homoscedasticity was assessed for continuous variables, which were then standardized using Z-scores. Group comparisons for continuous variables were executed through the ANOVA test with Bonferroni post hoc adjustment in the generalized linear model (GLM), encompassing data from the TUNEL test and the blastocyst morphology quality index. Additionally, the Kruskal-Wallis non-parametric test was employed to compare groups in terms of blastocoel, inner cell mass (ICM), and trophectoderm (TE) variables. The Chi-Square Test, comprising Pearson’s Chi-Square Test and Fisher’s Exact Probability, was utilized to assess group differences concerning cleavage, morula, and blastocyst rates. Significance for all tests was predetermined at p≤0.05.

## RESULTS

### Cleavage, morula, and blastocyst rates evaluation

The experiment was conducted in 8 replicates per group, resulting in 1645 cells collected, including zygote and oocytes. Among the embryos, 749 exhibited cleavage by day 2, and 316 developed to the blastocyst stage by day 5.

Regarding embryo cleavage rate in D2, the CONT-M group showed a significantly higher cleavage rate when compared to the other groups. On day 4, the CONT group showed a significantly higher morula formation rate in comparison with other groups.

Analysis of the blastocyst formation on day 5 revealed that the CONT-M group had a significantly higher blastocysts rate when compared to all other groups. A summary of these results is presented in Table 1.

**Table 1:** Embryo development according to SART classification, assessed by Chi-Square test.

		PCOS-M	PCOS	PLAC-M	PLAC	CONT-M	CONT	p
**Cleavage Rate D2**	Total Zygote/ Oocyte	408	388	312	301	122	114	**0.0001**
	Cleavage Embryos (N)	127	120	175	161	91	75	
	Cleavage Rate %	31.13%	30.93%	56.09%	53.49%	74.59%1	65.79%	
**Morula D4**	Cleavage Embryos (N)	127	120	175	161	91	75	**0.0001**
	Morula (N)	19	17	71	63	50	46	
	Morula Rate %	14.96%	14.17%	40.57%	39.13%	54.95%	61.33%2	
**Blastocyst D5**	Cleavage Embryos (N)	127	120	175	161	91	75	**0.0001**
	Blastocysts (N)	16	17	94	66	71	52	
	Blastocysts Rate %	12.60%	14.17%	53.71%	40.99%	78.02%^3^	69.33%	

1 Higher cleavage rate when compared to the other groups.

2 Higher morula rate when compared to the other groups.

3 Higher blastocyst rate when compared to the other groups. *p* values in bold indicates statistical significance (*p*≤0.0001).

### Blastocyst morphological assessment

**Supplementary Figure 1:**
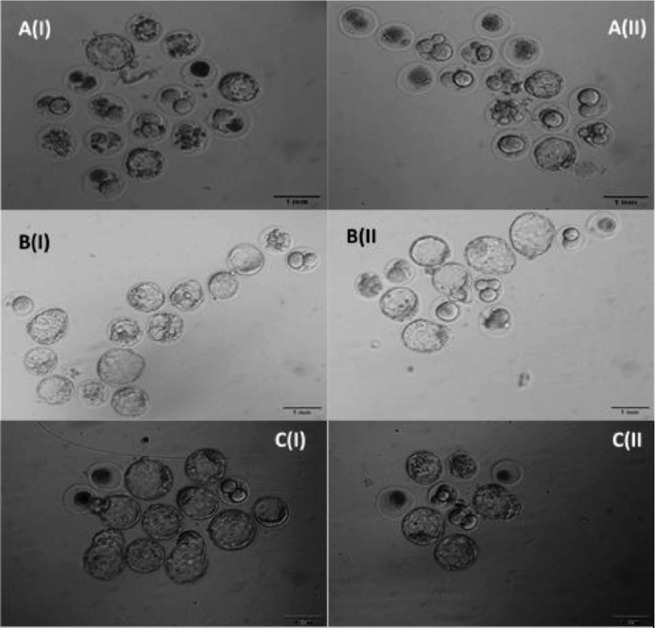
Inverted optical microscope micrograph at 100x magnification to evaluate embryo quality during the blastocyst stage. An (I) PCOS-M; A(II) PCOS; B(I) PLAC-M; B(II) PLAC; C(I) CONT-M; (II) CONT.

For this analysis, 316 blastocysts were evaluated (a representative image is shown in supplementary Figure 1). The quality index analysis revealed significant difference among PCOS, Placebo and Controls, independently of the use of melatonin (p=0.014, Table 2). Blastocysts from the PCOS-M and PCOS groups showed significantly lower quality index scores (7.3±1.66 and 7.5±1.50, respectively) in contrast to all other groups (PLAC-M, PLAC, CONT-M, and CONT), which had significantly higher scores (ranging from 13.2±0.90 to 14.3±1.21). For the morphological assessment (blastocoel, inner cell mass [ICM], and trophectoderm [TE] quality), the Kruskal-Wallis non-parametric test revealed significant differences among the groups in blastocoel quality (p=0.020) and trophectoderm quality (p=0.045), as shown in Table 3.

**Table 2:** Quality index assessment of the blastocyst morphology, by GLM comparison.

	PCOS-M (n=16) Mean±SD	PCOS (n=17) Mean±SD	PLAC-M (n=94) Mean±SD	PLAC (n=66) Mean±SD	CONT-M (n=71) Mean±SD	CONT (n=52) Mean±SD	p
Quality index	7.3±1.66^a^	7.5±1.50^a^	13.2±0.90^b^	14.3±1.21^b^	14.2±1.17^b^	13.7±1.42^b^	0.014

SD: Standard Deviation. *p* values in bold indicates statistical significance (*p*≤0.05).

a,b Different letters indicate statistical difference between the groups.

**Table 3:** Embryo quality assessment according with SART criteria.

	PCOS-M (n=16) Median; IR	PCOS (n=17) Median; IR	PLAC-M (n=94) Median; IR	PLAC (n=66) Median; IR	CONT-M (n=71) Median; IR	CONT (n=52) Median; IR	p
Blastocoel	3; 2.75	4; 2	4; 4	5; 3	5; 1	4; 1.75	**0.020**
ICM	1; 1	2; 1	2; 1	2; 1	2; 1	2; 1	0.063
TE	1; 1	1; 0.5	2; 1	2; 1	2; 1	2; 1	**0.045**

ICM: Inner cell mass; TE: Trophectoderm. *p* values in bold indicates statistical significance (*p*≤0.05). IR: Interquartile Range.

### TUNEL assay in blastocysts

**Figure 2:**
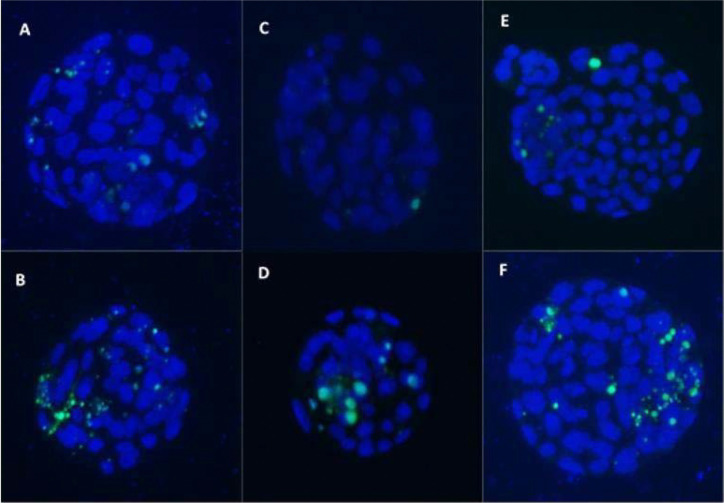
Inverted fluorescein microscope micrograph at 100x magnification of embryos that underwent the TUNEL assay. The cells in blue represent all blastocyst cells stained by DAPI and the green cells are indicating apoptosis (stained by FITC). (A) PCOS-M; (B) PCOS; (C) PLAC-M; (D) PLAC; (E) CONT-M; (F) CONT.

A total of 142 blastocysts were analyzed for apoptotic fragmentation using the TUNEL assay (Figure 2). A GLM was applied to compare the total cell number, number of apoptotic cells, and the apoptotic cell ratio (apoptotic cells/total cells) across experimental groups (Table 4). Significant differences were observed for both the total cell number and the apoptotic cell ratio (p=0.0001 for both), whereas the absolute number of apoptotic cells did not differ significantly among groups (p=0.359).

**Table 4:** Descriptive data from TUNEL assay in blastocysts, compared by GLM.

TUNEL	PCOS-M (n=11) Mean±SD	PCOS (n=13) Mean±SD	PLAC-M (n=33) Mean±SD	PLAC (n=32) Mean±SD	CONT-M (n=31) Mean±SD	CONT (n=22) Mean±SD	p
Total cells number (T)	40.4±5.81^a^	37.7±3.36^c^	59.2±3.60^de^	63.2±3.86b^de^	78.4±2.81^bdf^	58.5±3.04^de^	**0.0001**
Total apoptotic cells number (A)	5.9±1.41	4.6±0.94	3.7±0.39	4.0±0.43	4.8±0.61	4.9±0.79	0.359
Apoptosis ratio (A/T, %)	16.5±3.61^a^	11.9±2.14^a,b^	6.6±0.76^b^	7.1±0.85^b^	6.2±0.78^b^	8.4±1.33^b^	**0.0001**

a, b, c, d, e, f Different letters indicate statistical difference among the groups. *p* values in bold indicate statistical significance (*p*≤0.05).

The PCOS-M and PCOS groups exhibited significantly lower total cell numbers (40.4±5.81 and 37.7±3.36, respectively) compared to all other groups. In contrast, the CONT-M group had the highest total cell number (78.4±2.81), differing significantly from the PCOS groups and all other conditions. Similarly, the apoptotic cell ratio was markedly higher in the PCOS-M group (16.5±3.61%), followed by the PCOS group (11.9±2.14%), both showing significantly increased apoptosis compared to the placebo and control groups (ranging from 6.2±0.78% to 8.4±1.33%).

## DISCUSSION

PCOS patients show dysregulation of the hypothalamic-pituitary-ovarian axis, impairing folliculogenesis and leading to numerous small, underdeveloped follicles (Liao et al., 2021). This results in anovulatory infertility with a high follicle count but a small number of high-quality oocytes (Hariton et al., 2021).

The inflammation that plays a key role in expelling the MII oocyte from the follicle will also produce reactive oxygen species (ROS). In PCOS, high levels of ROS in serum and follicular fluid have been associated with decreased oocyte quality (Sayutti et al., 2022). High levels of ROS may result in the upregulation of interleukins and nitric oxide synthase 2, which can impair oocyte maturation (Schmidt et al., 2014). Additionally, ROS can lead to alterations in mitochondrial morphology, resulting in aneuploidy (Qiao & Feng, 2011). In our study, although ROS production was not assessed, we observed poor embryo quality in PCOS groups when compared to the other groups, regardless of melatonin supplementation. Melatonin had an important effect in controls, considering that CONT-M group had significant cleavage rates and blastocyst rates when compared to the other groups.

According to the literature, an increase of blastomere quantity is expected in all groups supplemented with melatonin (Liao et al., 2021; Zhang et al., 2022). Similarly, we observed that the use of melatonin influenced cleavage and morula development across all groups. It is noteworthy that when analyzing the blastocyst rate for PCOS, the use of melatonin did not improve these rates.

For the embryo quality assessment, PCOS embryos were of significantly poorer quality when compared to placebos and controls. In addition, while PCOS and Placebos had worse quality index when associated with melatonin supplementation, the control group had higher quality index related to the use of melatonin. Blastocoel and trophectoderm development were significantly lower in PCOS group, independent of melatonin supplementation. The significant lower blastocyst quality and the delay on embryo development in PCOS group may be a result of poor oocyte quality, which has been associated with granulosa cells dysfunction (Choi et al., 2008; Zhao et al., 2023). During oocyte differentiation, glucose and pyruvate are obtained from granulosa cells for energy and homeostasis (Sutton-McDowall et al., 2010). In the case of PCOS, granulosa cells have shown disturbances in mitochondrial activity and glycolysis, resulting in low oocyte competence (Zhao et al., 2023).

Although underlying mechanism of ROS in granulosa cells and subsequent fertilization are unknown, oxidative stress has been associated with high DNA fragmentation in granulosa cells from PCOS patients, followed by significant lower rates of good embryos formation in this group (Karuputhula et al., 2013). In embryos, oxidative stress results in DNA fragmentation and causes a slower embryo development or may even arrest it (Choi et al., 2008). When analyzing apoptosis results, the total cells number was significantly lower in PCOS group. Within this group, melatonin seems to increase the number of cells in embryos. Similarly, blastocysts from controls supplemented with melatonin also showed a higher number of cells when compared with controls without supplementation.

Melatonin activates the superoxide dismutases 2 (Sirt1/Sod2) pathway in granulosa cells, which seems to eliminate excess of ROS. In oocytes and granulosa cells melatonin has been reported to act as an antioxidant by upregulating B-cell lymphoma-2 gene (BCL-2) and downregulating B-cell lymphoma-2-associated X (BAX) and caspase-3, protecting oocytes from apoptosis, including in PCOS (Zhao et al., 2016). Moreover, the antioxidant role of melatonin has been related to upregulation of genes associated with steroidogenesis and angiogenesis, improving blood supply and therefore oocyte quality (Yu et al., 2019). According to the literature, these findings have potential to improve deficient oocytes phenotypes due to maternal aging and may therefore be used for the treatment of female infertility (Choi et al., 2008).

In embryos, melatonin binds to MT1 receptors that are expressed in blastocysts, resulting in a decrease in downstream molecules such as cAMP, cGMP and PLC. These molecules are linked to the mediation of physiological functions, closely related to lipidic expression; lipids are prone to undergo peroxidation, which partly leads to apoptosis. The outcome is that melatonin has a key role in supporting embryo development (Gao et al., 2012; Sampaio et al., 2012). In our study, melatonin seems to have a slight effect on the control group, due to the decreased apoptosis ratio when comparing CONT-M group to CONT group, although it is not significant. Overall, both PCOS groups had an increased apoptosis ratio, regardless of melatonin use. Other studies in embryos found that melatonin significantly promoted in vitro development of murine embryos, which reflected on blastocyst development and pregnancy rates. These effects have also been associated with antioxidant activity of melatonin by upregulation of SOD2 (Wang et al., 2013).

The literature includes few studies about the use of melatonin in culture media for oocytes and embryos. However, recent data shows that the use of melatonin in human embryos, from patients without specific infertility conditions, had an effect on embryo development on day 3 (Bao et al., 2022). Similarly, our study found an effect of melatonin in cleavage, including the PCOS group. Considering the antioxidant activity, the same study found an upregulation in catalase expression, without significant changes in other antioxidant gene expressions or reactive oxygen species (ROS) levels (Bao et al., 2022). With a different experimental design, another study shows that the women treated with Myo-inositol (MI) plus melatonin and vitamin D3 may benefit from this support to improve oocyte and blastocyst quality (Wdowiak & Filip, 2020). Our study used 10-6 mol/L (1µM) melatonin based on the range of melatonin concentration used in prior studies (Reiter et al., 2009; Tamura et al., 2022). This concentration seems to be on an effective range for antioxidant and cytoprotective effects in embryo culture, mainly in reproductive models to improve embryo morphology, reduce ROS, enhance mitochondrial function, and lower apoptosis without toxicity (Tamura et al., 2022). However, the doses of melatonin in different studies vary (Reiter et al., 2009) and future standardization would be crucial to evaluate its effects.

Melatonin supplementation in embryo culture did not significantly impact embryo quality in PCOS groups, however, both groups showed a higher number in apoptotic and total cell ratio in comparison to placebo and control groups. The PCOS-M group presents a lower blastocyst rate in comparison to the PCOS group. Under different experimental conditions, melatonin supplementation in IVF culture media seemed to improve the number of embryos in blastocyst stage (Navid et al., 2023). Additionally, the antioxidant potential was confirmed by the increase of expression level of the anti-apoptotic gene Bcl2, and significant decrease of the proapoptotic gene (Navid et al., 2023). However, it is important to consider that these findings are related to simultaneous inclusion of melatonin and vitamin C in the IVF medium.

For the placebos, although the supplementation with melatonin is associated with a lower number of total cells, it was also related to a lower number of apoptotic cells. Nonetheless, the ratios in placebo groups were not significant, which indicates that the effect of melatonin is inconclusive within these groups. Surprisingly, melatonin showed highest effects in blastocysts from controls, in which we observed a higher number of blastomere and the lowest number of apoptotic cells among all groups. These findings are in line with what is expected of melatonin capacity of regulating anti-apoptotic genes along with its antioxidant properties (Gao et al., 2012; Wdowiak & Filip, 2020).

Our results may be related to the melatonin concentration used in embryo culture. The concentration choice was listed as efficient for normal conditions and for non-PCOS with high oxidative stress environment mice embryos (Ishizuka et al., 2000). Additionally, when considering the use of three different melatonin concentrations (10-6 mol/L; 10-9 mol/L and 10-12 mol/L), the cleavage, morula and blastocyst rates were the highest for melatonin at 10-12 mol/L, meanwhile melatonin at 10-9 mol/L had the lowest apoptotic cell rate. On the other hand, the cleavage, morula and blastocyst rates for melatonin at 10-6 mol/L were the lowest when compared with the ones obtained using the other two concentrations (Ishizuka et al., 2000). Also, melatonin at 10-6 mol/L showed a higher apoptotic rate than melatonin at 10-9 mol/L (Ishizuka et al., 2000).

PCOS embryos may benefit from lower concentrations of melatonin to achieve better development and quality. Melatonin has been shown to improve the dysregulation of oxidative stress homeostasis associated with PCOS, besides playing other key roles in oocyte and embryo development (Jiang et al., 2021). However, in our study melatonin supplementation at 10-6 mol/L in the embryonic culture medium influenced embryo development in all groups, although apoptosis prevention was exclusive of the controls, supporting embryo development and preventing apoptosis. While our study discusses crucial insights about melatonin potential in embryo quality in PCOS, most studies focus on oocyte quality (Chang et al., 2014; Zou et al., 2020). Further exploration of the optimal dosage of melatonin is needed to assess its impact in embryo development and develop potential strategies of supplementation that would benefit embryo development.

## Data Availability

The data that support this study are available in FigShare at doi: 10.6084/m9.figshare.26072812.
